# Extent of intrinsic disorder and NMR chemical shift assignments of the distal N-termini from human TRPV1, TRPV2 and TRPV3 ion channels

**DOI:** 10.1007/s12104-022-10093-4

**Published:** 2022-06-06

**Authors:** Christoph Wiedemann, Benedikt Goretzki, Zoe N. Merz, Frederike Tebbe, Pauline Schmitt, Ute A. Hellmich

**Affiliations:** 1 Faculty of Chemistry and Earth Sciences, Institute of Organic Chemistry and Macromolecular Chemistry, Friedrich Schiller University Jena, Humboldtstraße 10, 07743 Jena, Germany; 2 Centre for Biomolecular Magnetic Resonance (BMRZ), Goethe University, Max von Laue Str. 9, 60438 Frankfurt, Germany; 3 Department of Chemistry, Division Biochemistry, Johannes-Gutenberg-University Mainz, Johann-Joachim Becher-Weg 30, 55128 Mainz, Germany

**Keywords:** Transient receptor potential, TRP vanilloid, Ion channel, Intrinsically disordered protein, Regulatory domain, Structural dynamics

## Abstract

The mammalian Transient Receptor Potential Vanilloid (TRPV) channels are a family of six tetrameric ion channels localized at the plasma membrane. The group I members of the family, TRPV1 through TRPV4, are heat-activated and exhibit remarkable polymodality. The distal N-termini of group I TRPV channels contain large intrinsically disordered regions (IDRs), ranging from ~ 75 amino acids (TRPV2) to ~ 150 amino acids (TRPV4), the vast majority of which is invisible in the structural models published so far. These IDRs provide important binding sites for cytosolic partners, and their deletion is detrimental to channel activity and regulation. Recently, we reported the NMR backbone assignments of the distal TRPV4 N-terminus and noticed some discrepancies between the extent of disorder predicted solely based on protein sequence and from experimentally determined chemical shifts. Thus, for an analysis of the extent of disorder in the distal N-termini of all group I TRPV channels, we now report the NMR assignments for the human TRPV1, TRPV2 and TRPV3 IDRs.

## Biological Context

Transient Receptor Potential (TRP) channels are tetrameric cation channels involved in temperature and pain sensation, nociception and osmoregulation (Ramsey et al. 2006). They are classified into subfamilies primarily based on amino acid sequence and structural characteristics rather than functional properties (Himmel and Cox 2020). Members of the mammalian TRPA (ankyrin), TRPC (canonical), TRPM (melastatin) and TRPV (vanilloid) subfamilies are localized at the plasma membrane and feature extensive N- and C-terminal cytoplasmatic domains. The mammalian TRPML (mucolipin) and TRPP (polycystin) proteins possess a large domain located between the first two transmembrane helices (Hellmich and Gaudet 2014; Samanta et al. 2018; Viet et al. 2019).

The mammalian TRPV subfamily can be further subdivided into group I and group II channels. TRPV1 through TRPV4 (group I) are activated by heat and, together with temperature-sensitive members of other TRP channel subfamilies, constitute the so-called “ThermoTRPs” (Cohen and Moiseenkova-Bell 2014; Islas 2017). Human group I TRPV channels are widely expressed, e.g. in primary sensory neurons, dorsal root ganglia, trigeminal ganglia, testis, spleen, intestine, bladder, brain, heart, liver, endothelia, and many other tissues (Cortright et al. 2001; Toledo Mauriño et al. 2020). In addition to thermosensation, they generally exhibit remarkable polymodality, i.e. the ability to respond to very diverse and seemingly unrelated stimuli such as natural and artificial compounds, pH, ions, small molecules or lipids (Baez-Nieto et al. 2011). Notably, many active ingredients from spices and herbs activate group I TRPV channel members, e.g. capsaicin (from chili) (Caterina et al. 1997), piperine (pepper) (Dong et al. 2019), allicin (garlic) (Macpherson et al. 2005) for TRPV1 or thymol (thyme) and carvacrol (oregano) (Xu et al. 2006) for TRPV3. Group I TRPV channels also play important roles in human health, e.g., in cancer, the immune system, viral infections or neurodegenerative diseases (Prevarskaya et al. 2007; Nilius and Voets 2013; Omar et al. 2017; Bujak et al. 2019; Taga et al. 2022). In contrast to the stimuli-dependent, non-selective group I TRPV calcium channels, the group II members, TRPV5 and TRPV6, are constitutively active and calcium-selective (van Goor et al. 2020).

To date, high-resolution cryo-electron microscopy (cryo-EM) structures have been determined for all six mammalian TRP vanilloid members (e.g. TRPV1 (Liao et al. 2013; Gao et al. 2016), TRPV2 (Huynh et al. 2016; Pumroy et al. 2019), TRPV3 (Singh et al. 2018, 2019; Zubcevic et al. 2018, 2019; Nadezhdin et al. 2021), TRPV4 (Deng et al. 2018), TRPV5 (Hughes et al. 2018; Dang et al. 2019) and TRPV6 (McGoldrick et al. 2018)). These structures demonstrate a significant 3D structural conservation within the transmembrane region of these channels. In addition, all TRPV channels contain an N-terminal ankyrin repeat domain (ARD). However, while TRPV5 and TRPV6 proteins feature an N-terminal α-helix preceding the ARD (e.g. Hughes et al. 2018; McGoldrick et al. 2018), the distal cytosolic N-termini of group I TRPV channels contain intrinsically disordered regions (IDRs) of variable length: ~100 aa for TRPV1, ~ 75 for TRPV2, ~ 120 for TRPV3, and ~ 150 for TRPV4. To date, structural studies could not capture most of these regions critical for protein function and regulation. Nonetheless, the group I TRPV channel IDRs act as important interaction platforms for cytosolic binding partners and post-translational modifications (Voolstra and Huber 2014; Goretzki et al. 2018; Aisenberg et al. 2022) and they play an important role in stimulus-dependent channel activation (e.g. Liao et al. 2013; Zubcevic et al. 2016; Deng et al. 2018; Shimada et al. 2020).

Given the general lack of structural data available for the distal N-terminus in the majority of group I TRPV channel structures, many questions regarding the structural coupling of these regions to the channel pore, and the conformational propagation of peripheral ligand binding remain unanswered. We have previously described the NMR backbone assignments of the human and chicken TRPV4 IDR with 147 and 133 amino acids, respectively (Goretzki et al. 2022). Now, to extend the structural and dynamic analysis of the IDRs to the remaining members of the human group I TRPV channels, we report the assignments of the N-terminal IDRs of the human TRPV1 (UniProtKB: Q8NER1-1, residues K2-D100, 99 amino acids), TRPV2 (UniProtKB: Q9Y5S1-1, residues T2-R73, 72 aa), and TRPV3 (UniProtKB: Q8NET8-1, residues K2-L119, 118 aa) ion channels.

## Methods and experiments

### Protein expression and purification

The DNA sequences encoding the *H. sapiens* N-terminal TRPV1, TRPV2 and TRPV3-IDRs (hsTRPV1-IDR, hsTRPV2-IDR and hsTRPV3-IDR, respectively) with an N-terminal His_6_-SUMO-tag cloned into a pET11a vector were obtained from Genescript (NJ, USA).

To obtain uniformly ^13^C, ^15^N-labeled hsTRPV1-IDR, hsTRPV2-IDR and hsTRPV3-IDR, transformed *Escherichia coli* BL21-Gold(DE3) cells (Agilent Technologies) were grown in M9 minimal medium supplemented with 0.1 mg/mL Ampicillin, 1 g/l ^15^NH_4_Cl and 2 g/l ^13^C_6_-labeled glucose as the sole nitrogen and carbon sources. Gene expression was induced at an OD_600nm_ of 0.8-1.0 by adding 1 mM IPTG (isopropyl-1-β-d-galactopyranoside). Cells were grown overnight at 16 °C, harvested via centrifugation (4500xg, 20 min, 4 °C) and stored at -80 °C until further use.

For protein purification, cells were resuspended in lysis buffer (50 mM NaP_i_ pH 7.5, 300 mM NaCl, 5 mM imidazole, 1 mM PMSF, 1 mM benzamidine, lysozyme, DNAse, RNAse, and protease inhibitor (cOmplete Mini, Roche Diagnostics GmbH)). For hsTRPV3-IDR, DTT was added to a final concentration of 1 mM in all purification buffers. Resuspended cells were sonicated on ice (Bandelin SONOPULS) and cell debris was removed by centrifugation (20000xg, 20 min, 4 °C). The supernatants were loaded onto pre-equilibrated gravity flow Ni-NTA (Qiagen) affinity columns at 4 °C. After washing twice (50 mM NaP_i_ pH 7.5, 300 mM NaCl, 5 mM imidazole, then 10 mM imidazole), proteins were eluted in one column volume fractions with 50 mM NaP_i_ pH 7.5, 300 mM NaCl, 250 mM imidazole. Protein containing fractions were pooled and dialyzed overnight (50 mM NaP_i_ pH 7.5, 300 mM NaCl, 1 mM DTT) in the presence of 5 mol% Ulp-1 protease at 4 °C. Reverse Ni-NTA affinity chromatography was employed for separation of cleaved proteins from His_6_-SUMO-tag containing constructs.

Subsequently, size exclusion chromatography (HiLoad prep grade 10/300 Superdex75 column (GE Healthcare)) using 10 mM Tris-HCl pH 7, 100 mM NaCl (hsTRPV1-IDR, hsTRPV2-IDR) or 10 mM NaP_i_ pH 6.2, 300 mM NaCl, 1 mM DTT (hsTRPV3-IDR) was carried out. Sample purity was verified by SDS-PAGE. The purified, tag-free proteins were frozen in liquid N_2_ and stored at -80 °C until further use.

Of note, the first residue (M1) was missing in the final constructs used for NMR experiments. Thus, the constructs comprise residues K2-D100 in hsTRPV1-IDR, T2-R73 in hsTRPV2-IDR and K2-L119 in hsTRPV3-IDR, respectively.

### NMR spectroscopy

All NMR spectra of human TRPV1-IDR, TRPV2-IDR and TRPV3-IDR were recorded at 25 °C on 600 MHz Bruker AvanceIII HD NMR spectrometer systems equipped with cryogenic triple resonance probes (Bruker Biospin GmbH, Rheinstetten, Germany). The spectrometers were locked on D_2_O.

The ^1^H chemical shifts of the ^13^C,^15^N-labelled proteins were directly referenced to 3-(trimethylsilyl)propane-1-sulfonate (DSS). ^13^C and ^15^N chemical shifts were referenced indirectly to the ^1^H DSS standard by the magnetogyric ratio (Wishart et al. 1995).

Backbone and side chain chemical shift resonances were assigned with a set of band-selective excitation short-transient (BEST) (Schanda et al. 2006) transverse relaxation-optimized spectroscopy (TROSY)-based (Pervushin et al. 1997) experiments: [^1^H,^15^N]-TROSY, HNCO, HN(CA)CO, HNCA, HNCACB (Schulte-Herbrüggen and Sørensen 2000; Lescop et al. 2007; Favier and Brutscher 2011; Solyom et al. 2013). In addition, HBHA(CO)NH spectra (Grzesiek and Bax 1993; Muhandiram and Kay 1994) were recorded for proton resonance assignments of hsTRPV1-IDR and hsTRPV2-IDR. For hsTRPV3-IDR, ^13^C and ^1^H side chain chemical shift information was obtained from (H)CC(CO)NH (Montelione et al. 1992; Grzesiek et al. 1993) and [^1^H,^15^N]-TOCSY-HSQC spectra (Marion et al. 1989).

All spectra were processed using Bruker Topspin 3.6.2 or 4.1.1 and analyzed using CARA (www.cara.nmr.ch) or CcpNmr Analysis (Vranken et al. 2005) v2.4 (locally installed) or v2.5 (within the NMRbox virtual environment (Maciejewski et al. 2017)).

### Disorder prediction

To predict the structural disorder of the N-terminal regions of the human TRPV1, TRPV2 and TRPV3 channels based on their primary structures, we used the ODiNPred web server (https://st-protein.chem.au.dk/odinpred) (Nielsen and Mulder 2019; Dass et al. 2020).

Further, the POTENCI tool ( https://st-protein02.chem.au.dk/potenci) (Nielsen and Mulder 2018) was used to calculate the random coil chemical shifts for hsTRPV1-IDR, hsTRPV2-IDR, and hsTRPV3-IDR based on their respective amino acid sequence at our experimental conditions (temperature, pH value and ionic strength). Using the backbone NMR assignments, we additionally applied the SSP program (Marsh et al. 2006) to assess the amount of secondary structure formation within the IDRs of the three TRPV channels.

## Extent of assignment and data deposition

Although detailed structural information on TRPV channels is available from X-ray and cryo-EM studies, in most cases the distal N-terminal region preceding the ARD escaped description at an atomic level, either because it was purposefully deleted or because it remained unresolved due to its inherent flexibility (Goretzki et al. 2021). With a spectroscopic approach, we recently showed that the N-terminal regions of human and chicken TRPV4 channels are almost completely disordered (Goretzki et al. 2018, 2022). Interestingly, this was somewhat contradicted by sequence-based disorder prediction web tools that indicated the presence of a significant amount of secondary structure propensity in the TRPV4 IDRs (Goretzki et al. 2022).

**Fig. 1:**
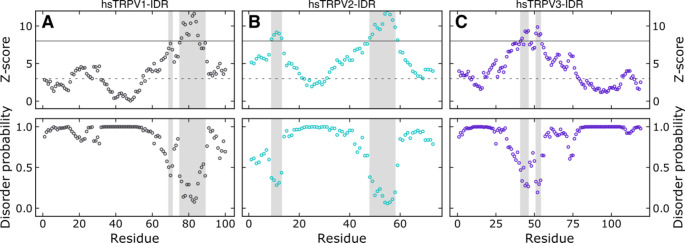
The sequence-based ODiNPred analysis predicts a significant amount of disorder in the distal N-termini of the human TRPV1, TRPV2 and TRPV3 channels. Regions with low predicted disorder propensities (shaded in light grey), as seen in particular for TRPV1 and TRPV2, could indicate the transient formation of structural order. The circles show the residue-specific Z-score (upper panel) and disorder probability (lower panel). A residue specific Z-score larger than 8 (solid line) indicates structural order while a Z-score below three (dashed line) predicts full disorder. Z-scores between three and eight reflect transient local structure propensity. The Z-score and disorder probability were calculated using the ODiNPred webserver (Dass et al. 2020)

Here, to estimate the potential flexibility and disorder of the N-termini of the three remaining group I human TRPV channels and to compare this to NMR spectroscopic data, we used the sequence-based web server ODiNPred (Dass et al. 2020) (Fig. 1). ODiNPred predicts disorder probabilities larger than 0.5 for 86% of residues within TRPV1-IDR, 78% of residues within TRPV2-IDR and 91% of residues within TRPV3-IDR, which is indicative of highly disordered proteins. Nonetheless, on the C-terminal end of the TRPV1- and TRPV2-IDRs, lower disorder probabilities (< 0.5) are predicted for continuous stretches of amino acids in 14 of 100 and 16 of 72 residues, respectively. Such values are indicative of transient structural order within ensembles sampling the potential conformational space. In addition, in the N-terminal part of hsTRPV2-IDR, a second short region with low per-residue disorder probability is predicted. Notably, in hsTRPV3-IDR only 10 of 109 residues show a disorder probability lower than 0.5.

**Fig. 2:**
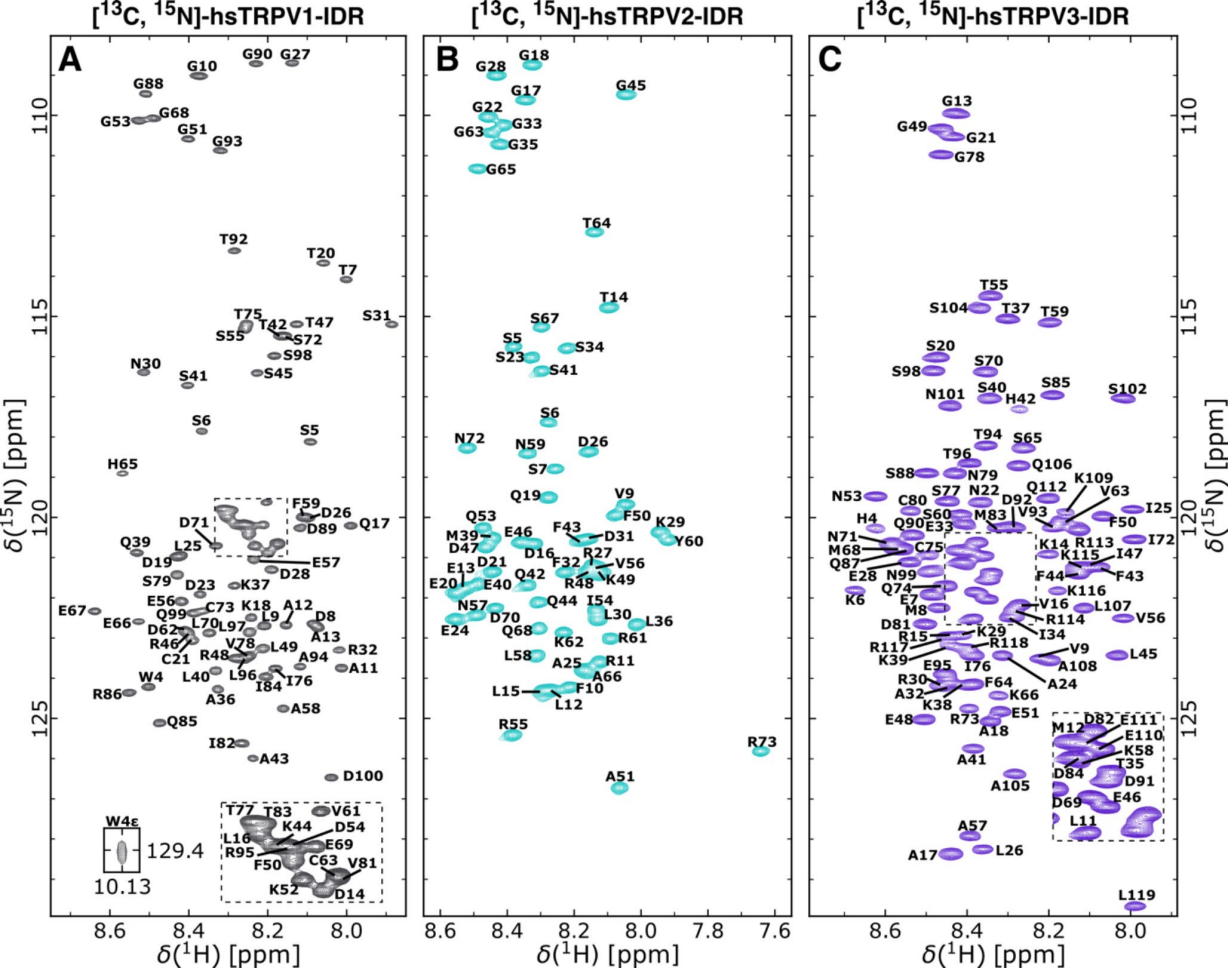
[^1^H, ^15^N]-TROSY-HSQC spectra of ^13^C, ^15^N-labeled human TRPV1-IDR (residues 2-100) (A), TRPV2-IDR (residues 2–73) (B) and TRPV3-IDR (residues 2-119) (C) recorded at 600 MHz and 25 °C. Human TRPV1-IDR and TRPV2-IDR were measured in 10 mM Tris-HCl pH 7, 100 mM NaCl and hsTRPV3-IDR was measured in 10 mM NaPi pH 6.2, 300 mM NaCl, 1 mM DTT. 10% D_2_O for field-frequency locking and 0.1 mM DSS for internal ^1^H chemical shift referencing was added to all samples. Assigned residues are annotated in single letter amino acid code according to the human TRPV1 (UniProtKB: Q8NER1-1), TRPV2 (UniProtKB: Q9Y5S1-1), and TRPV3 (UniProtKB: Q8NET8-1) protein sequences.

To experimentally characterize the N-terminal regions of the human TRPV1, TRPV2 and TRPV3 channels in solution, we used NMR spectroscopy. In agreement with a predicted low overall secondary structure content, the [^1^H, ^15^N]-TROSY-HSQC-spectra of human TRPV1-IDR, hsTRPV2 and TRPV3-IDR show limited signal dispersion in the ^1^H^N^ dimension (Fig. 2 A-C), indicating a similar chemical environment of all ^1^H^N^ nuclei and an inherent lack of stable structural elements. By using a set of two- and three-dimensional NMR experiments, the sequence specific resonance assignments for nearly all backbone ^1^H, ^13^C and ^15^N spins could be obtained. In summary, 91.3%, 92.6% and 91.1% of the H^N^, N´, C´, C^α^, C^β^, H^α^ and H^β^ resonances of hsTRPV1-, hsTRPV2- and hsTRPV3-IDR could be assigned, respectively.

All three proteins contain a large number of proline residues, i.e., 14 in TRPV1-IDR, 7 in TRPV2-IDR and 17 in TRPV3-IDR. These do not group in extended proline rich regions as in the N-terminus of TRPV4, but nonetheless frequently cluster in pairs of two or three. For the human TRPV1-IDR, the C´, C^α^, C^β^ resonances for 12 out of 14 proline residues could be assigned with mean C^β^ values of 32.17 ± 0.18 ppm. This leaves only the consecutive proline residues in the triple proline repeat P33*-P34*-P35 unassigned (marked with *).

Likewise, backbone carbon chemical shifts for 6 out of 7 proline residues in hsTRPV2-IDR were assigned with mean C^β^ values of 32.12 ± 0.06 ppm, leaving residue 37 in the P37*-P38 double proline motif unassigned.

For the hsTRPV3-IDR, the C´, C^aα^, C^β^ as well as C^γ^ resonances for 16 of 17 proline residues could be assigned, leaving only P61 in the P61*-P62 motif without chemical shift information. All assigned hsTRPV3-IDR prolines show ^13^C^β^ and ^13^C^γ^ values in the range of 32.17 ± 0.08 ppm and 27.47 ± 0.16 ppm, respectively. The mean difference of the proline ^13^C^β^ and ^13^C^γ^ chemical shifts is 4.69 ± 0.20 ppm.

Based on the chemical shift values for C^β^ and C^γ^, it can be assumed that all assigned proline residues in hsTRPV1-IDR, hsTRPV2-IDR and hsTRPV3-IDR are in the *trans* configuration (Schubert et al. 2002; Shen and Bax 2010). It remains to be seen whether these residues can adopt a stable *cis* configuration in the presence of ligands, as was observed for TRPV4 (Goretzki et al. 2018).

**Fig. 3:**
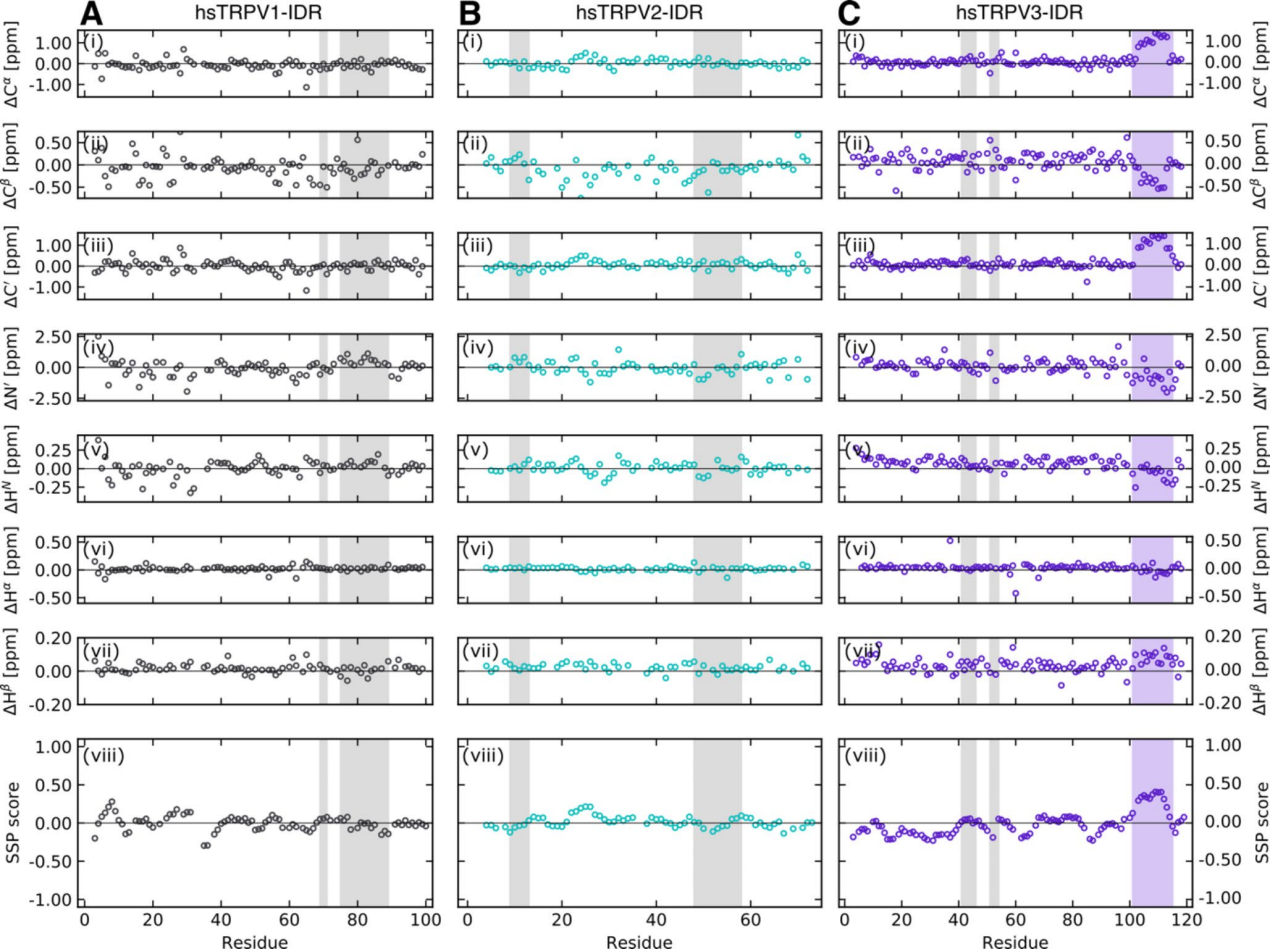
Secondary chemical shifts analysis reveals that the distal N-termini of the human TRPV1, TRPV2 and TRPV3 channels are highly disordered. (i-vii) Secondary chemical shifts are calculated as the differences between the experimentally determined and predicted C^α^, C^β^, C´, N´, H^N^, H^α^, H^β^ chemical shifts. POTENCI (Nielsen and Mulder 2018) was used for sequence-based prediction of random coil chemical shifts. (viii) Secondary structure propensity (SSP) prediction based on chemical shifts using the SSP script (Marsh et al. 2006). A positive and negative SSP score reflects α-helix and β-sheet propensities, respectively. A SSP value of 1 reflects a fully formed helical-structure and a value of -1 a fully formed βstrand, respectively. Only ^13^C^α^, ^13^C^β^ and ^1^H^α^ chemical shifts of non-proline preceding residues were used when running the SSP script. For better comparison with Fig. 1, the regions with low predicted disorder propensities based on ODiNPred are shaded in light grey. The stretch of residues (P103-R113) in the C-terminal region of the hsTRPV3-IDR potentially forming an α-helical element is highlighted in light purple. Of note, ODiNPred does not predict secondary structure formation for this region in TRPV3 (Fig. 1 C)

The experimentally obtained chemical shifts of the distal N-termini of hsTRPV1, hsTRPV2 and hsTRPV3 were also used for an initial secondary chemical shift-based structural analysis. The POTENCI web server (Nielsen and Mulder 2018) was used for the prediction of random coil chemical shifts at our experimental conditions. The predicted chemical shifts were compared with those experimentally obtained to reveal potential regions of structural order. For all three TRPV channel constructs, the measured and predicted C^α^, C^β^, C´, N´, H^*N*^, H^α^, and H^β^ chemical shift values agree remarkably well (Fig. 3 A-C, i-vii). The mean differences between the experimental and POTENCI-predicted random coil chemical shift values for hsTRPV1-IDR are: ΔC^α^ = -0.08 ± 0.24 ppm, ΔC^β^ = -0.08 ± 0.27 ppm, ΔC´ = 0.00 ± 0.26 ppm, ΔN´ = -0.03 ± 0.64 ppm, ΔH^N^ = 0.01 ± 0.10 ppm, ΔH^α^ = 0.02 ± 0.05 ppm, and ΔH^β^ = 0.02 ± 0.03 ppm. Likewise, the mean differences for hsTRPV2-IDR are: ΔC^α^ = -0.02 ± 0.17 ppm, ΔC^β^ = -0.09 ± 0.22 ppm, ΔC´ = 0.06 ± 0.18 ppm, ΔN´ = -0.03 ± 0.51 ppm, ΔH^N^ = 0.01 ± 0.07 ppm, ΔH^α^ = 0.02 ± 0.04 ppm, and ΔH^β^ = 0.02 ± 0.02 ppm. For hsTRPV3-IDR the mean differences between experimental and predicted chemical shifts are: ΔC^α^ = 0.18 ± 0.36 ppm, ΔC^β^ = 0.06 ± 0.21 ppm, ΔC´ = 0.20 ± 0.38 ppm, ΔN´ = 0.00 ± 0.61 ppm, ΔH^N^ = 0.06 ± 0.09 ppm, ΔH^α^ = 0.03 ± 0.11 ppm, and ΔH^β^ = 0.04 ± 0.04 ppm.

The notion that the human TRPV1, TRPV2 and TRPV3 distal N-termini are highly disordered is further supported by the sequence-specific secondary structure propensity method (Marsh et al. 2006). As recommended for intrinsically disordered proteins, we used the SSP method to combine C^α^, C^β^ and H^α^ chemical shift values into single residue specific scores (Fig. 3 A-C, viii). In contrast to the analysis with the ODiNPred server, which indicated that hsTRPV1-IDR and hsTRPV2-IDR but not hsTRPV3-IDR contain extended regions able to form transient structures (Fig. 1), the SSP scores predict both the TRPV1 and TRPV2-IDR sequences to be highly disordered (mean SSP scores of 0.000 ± 0.097 and 0.015 ± 0.075 for hsTRPV1-IDR and hsTRPV2-IDR, respectively). Globally, the mean SSP score of the hsTRPV3-IDR is also close to 0 (-0.009 ± 0.156). However, a stretch of residues (P103-R113) in the C-terminal region of the hsTRPV3-IDR shows a significantly higher mean SSP score (0.355 ± 0.041), suggesting the potential formation of a helical structural element. The presence of an α-helical structure in this region is supported by recent cryo-EM structures of human TRPV3 (e.g. Zubcevic et al. 2019). Interestingly, AlphaFold (Jumper et al. 2021) also predicts a helical structure for this region.

By averaging the calculated SSP scores, an overall secondary structure content of only 7.7% for hsTRPV1-IDR, 6.0% for hsTRPV2-IDR and 12.0% for hsTRPV3-IDR can be estimated.

Although partial structural information on the N-terminal IDRs of group I TRPV channel members has been reported (e.g. Zubcevic et al. 2019; Pumroy et al. 2019; Nadezhdin et al. 2021), for the most part these regions are missing from structural studies. NMR spectroscopy is an optimal tool to investigate highly flexible and intrinsically disordered proteins. Together with our previous study on TRPV4 (Goretzki et al. 2022) we supplement the available structural information on group I TRPV channels with a detailed view on the extent of intrinsic disorder in their distal N-termini at atomic resolution. Interestingly, while there is some agreement with sequence-based structure predictions, the disorder determined *in vitro* tends to be more extensive than what is seen *in silico*. The inherent flexibility of the N-termini of group I TRPV channels is likely a conserved molecular feature underpinning their importance for channel regulation but making them challenging targets for structural studies.

## Data Availability

The backbone assignments of the wildtype human TRPV1, TRPV2 and TRPV3 N-terminal intrinsically disordered regions have been deposited in the BioMagResBank (https://bmrb.io) under the accession numbers 51353 (TRPV1-IDR, residues 2-100), 51354 (TRPV2-IDR, residues 2–73) and 51355 (TRPV3-IDR, residues 2-119).
